# Effect of WeChat-based intervention on food safety knowledge, attitudes and practices among university students in Chongqing, China: a quasi-experimental study

**DOI:** 10.1186/s41043-023-00360-y

**Published:** 2023-04-05

**Authors:** Zhengjie Cai, Xinmiao Luo, Xianglong Xu, Zumin Shi, Cesar Reis, Manoj Sharma, Xiaorong Hou, Yong Zhao

**Affiliations:** 1 Department of Nutrition and Food Hygiene, School of Public Health, Chongqing Medical University, No, 1 Yixueyuan Road, Yuzhong District, Chongqing, China; 2 Department of Health Behavior and Social Medicine, West China School of Public Health and West China Fourth Hospital, Sichuan University, Sichuan, China; 3 Songzi Center for Disease Control and Prevention, Jingzhou, Hubei China; 4 Central Clinical School, Faculty of Medicine, Nursing and Health Sciences, Monash University, Melbourne, Australia; 5 Melbourne Sexual Health Centre, Alfred Health, Melbourne, Australia; 6 China-Australia Joint Research Center for Infectious Diseases, School of Public Health, Xi’an Jiaotong University Health, Science Center, Xi’an, , Shaanxi China; 7 Human Nutrition Department, College of Health Sciences, QU Health, Qatar University, Doha, Qatar; 8 Department of Occupational Medicine, Kaiser Permanente-Southern California Medical Group, Los Angeles, USA; 9 Environmental and Occupational Health, School of Public Health, University of Nevada, Las Vegas, USA; 10 Department of School of Medical and Information, Chongqing Medical University, No. 1 Yixueyuan Road, Yuzhong District, Chongqing, China; 11 Research Center for Medicine and Social Development, Chongqing Medical University, Chongqing, China; 12 The Innovation Center for Social Risk Governance in Health, Chongqing Medical University, Chongqing, China; 13 Chongqing Key Laboratory of Child Nutrition and Health, Chongqing, China

**Keywords:** Food safety, Knowledge, Attitudes, Practices, Intervention, WeChat official account

## Abstract

**Background:**

Food safety is of global importance and has been of concern in university settings in recent years. However, effective methods to conduct food safety education are limited. This study aims to evaluate the effects of an intervention on food safety knowledge, attitudes and practices (KAP) by social media, WeChat, among university students.

**Methods:**

A quasi-experimental study was conducted in Chongqing, China. Two departments were recruited randomly from a normal university and a medical university. One department from each university was randomly selected as the intervention group and the other as the control group. All freshmen students in each selected department were chosen to participate in this study. One thousand and twenty-three students were included at baseline, and 444 students completed the study. This intervention was conducted through food safety-related popular science articles with an average of three articles per week released by WeChat official accounts called "Yingyangren" for two months to the intervention group. No intervention was conducted in the control group. An independent t-test was used to test statistical differences in the food safety KAP scores between the two groups. A paired t-test was used to test statistical differences in the food safety KAP scores between before and after the intervention. And quantile regression analysis was conducted to explore the difference between the two groups across the quantile levels of KAP change.

**Results:**

After the intervention, compared with control group, participants in the intervention group did not score significant higher on knowledge (*p* = 0.98), attitude (*p* = 0.13), and practice (*p* = 0.21). And the scores of food safety knowledge and practices slightly improved after the intervention both in the intervention group (*p* = 0.01 and *p* = 0.01, respectively) and in the control group (*p* = 0.0003 and *p* = 0.0001, respectively). Additionally, the quantile regression analysis showed that the intervention had no effect on improving the food safety KAP scores.

**Conclusions:**

The intervention using the WeChat official account had limited effects on improving the food safety KAP among the university students. This study was an exploration of food safety intervention using the WeChat official account; valuable experience can be provided for social media intervention in future study.

***Trial registration*:**

ChiCTR-OCH-14004861.

**Supplementary Information:**

The online version contains supplementary material available at 10.1186/s41043-023-00360-y.

## Introduction

Food safety is a global health goal. Foodborne diseases represent a growing public health problem in developed and developing countries [1]. Global estimates that 31 foodborne hazards cause 600 million foodborne illnesses and 420,000 deaths annually, resulting in the loss of 33 million healthy life years [2]. According to Foodborne Diseases Surveillance Network, a total of 2,401 foodborne diseases occurred and resulted in 21,374 cases and 139 deaths in the 29 provinces of mainland China in 2015 [3]. Moreover, cases of foodborne diseases were often under-reported, especially in developing countries [4].

Food safety has been of concern in university settings in recent years. University students are one of the high-risk population groups for food poisoning, who have inadequate knowledge [5–7] and risky food safety practices. University students typically eat out [8], consume takeaway food [9] and have unhealthy food handling [10]. Normal and medical university students belong to a population with unique features. Normal universities provide teacher education in China, in which various types of teachers were trained. Food safety cognition and practices of teachers and doctors are beneficial to their food safety incidence prevention and are expected to play important roles in health education and promotion after their graduation [6].

The World Health Organization stated that food safety education is vital in eliminating or reducing food contaminants and preventing micro-organism growth at levels that cause disease [11]. Some food safety intervention programs were conducted targeting food service employees [12–14] and students [15, 16], and traditional education methods were often used [17, 18]. These methods included providing reading materials (e.g. booklets and leaflets), conducting lectures and presentations and distributing posters [19]. A previous study demonstrated that methodology and approach adopted are important for a successful food safety training programme [20]. The limited effectiveness of traditional health education [21, 22] leads health education and promotion researchers worldwide to explore effective and innovative ways, which attempt to increase the efficacy of their interventions based on the worldwide web and other digital media [23].

One of the leading social networks worldwide, WeChat, developed by the Chinese company Tencent, placed fifth in the number of active users and had over 1.1 billion monthly active users in the first quarter of 2019 [24]. In accordance with the statistics provided by the China Internet Network Information Center in 2019 [25], the percentage of WeChat users reached 83.4% in China. Most of its users were between the ages of 20 and 29 by the end of December 2018. Like Facebook, Twitter and Instagram, WeChat offers a free instant messaging application for smartphones that enables the exchange of voice, text, pictures, videos and location information via mobile phone indexes [26]. WeChat official account is based on a new functional module. WeChat users can register an official account, which enables them to push feeds, interact with one another and provide subscribers with service. In addition, subscribers can read messages and communicate with others through these official accounts [27]. At present, WeChat, as a cost-effective and peer-to-peer supported educational tool, has been used for conducting health education or promotion to modify behaviours [27–30]. However, concerns exist about reliability and quality control of disseminated information via social media, as well as concerns about the intervention effects on promoting healthy behaviours [31–33]. Understanding the effect of WeChat on users is important as it gains popularity as a health intervention platform.

At present, most intervention strategies for improving food safety cognition and practices are mainly based on traditional education methods. Previous studies on social media and food safety were targeted at communication of food safety risks or public opinion on the Internet regarding food safety [27, 34, 35]. Intervention research on improving food safety cognition and practices via WeChat among university students is limited. Most university students acquired food safety knowledge through the Internet [36], which provided the foundation for conducting intervention to improve food safety knowledge, attitude and practices (KAP) by using WeChat amongst university students. Therefore, combined with our group’s experience with WeChat intervention design [37], this study aims to evaluate the effect of the intervention on food safety KAP by using a WeChat official account amongst university students.

## Method

### Study design

A quasi-experimental study was conducted to evaluate the effect of food safety KAP intervention using the WeChat official account amongst university students in Chongqing, China. We used a three-stage stratified cluster sampling method to recruit participants. Firstly, a normal and a medical university was selected in Chongqing University Town. Secondly, two departments were selected in each designated university. One department from each university was randomly selected as the intervention group and the other as the control group, with a 50% chance of being allocated to either group by using the coin-toss method. Lastly, all freshmen students in each selected department were chosen to participate in this study. Inclusion criteria were the following: (1) that all students participated in the study willingly and (2) the participants were users of WeChat (used the application more than once and more than an hour in the past week). Exclusion criteria were the following: (1) that students were unwilling to participate in the study and (2) the students did not use WeChat as described.

### Patient and public involvement

No patient involved.

### Baseline investigations

Before the intervention, all participants were asked to complete a baseline self-administered questionnaire, including demographic characteristics and food safety KAP. Details of the baseline questionnaire, survey method and pre-test have been reported [6].

### Intervention

The introduction of "Yingyangren" WeChat official account is shown in Additional file 1: Fig. 1. The "Yingyangren" WeChat official account (Additional file 1: Fig. 1a) was developed by our research team and a specialized information technology company, which was a relatively experienced WeChat media platform for delivering health knowledge. In this study, the WeChat official account was used to publish food safety-related popular science articles amongst the intervention group. People who followed the official account could read new messages (Additional file 1: Fig. 1b and 1c) and review the message history (Additional file 1: Fig. 1d) of content previously published on the official account. A consultation functional interface (Additional file 1: Fig. 1e and 1f) was open for communication with health experts. The research group was made up of four professors of nutrition and food hygiene and one professor of medical information. And several postgraduates participated in the design and drafting of food safety-related popular science articles, and all articles were reviewed by the research group members before publication on the "Yingyangren" WeChat official account. Moreover, all responses to the questions raised by users were evaluated by the research group members to ensure correctness and rationality.

Leaflets were made by our research team to explain the importance, objectives and methodology of the study to attract students’ active participation and attached to the 2D code of the "Yingyangren" WeChat official account. Our members propagated the WeChat official account platform amongst the intervention group and invited them to follow the account, which was used to release food safety-related popular science articles and disseminate food safety knowledge amongst the intervention group. The control group did not receive any propagation.

**Fig. 1:**
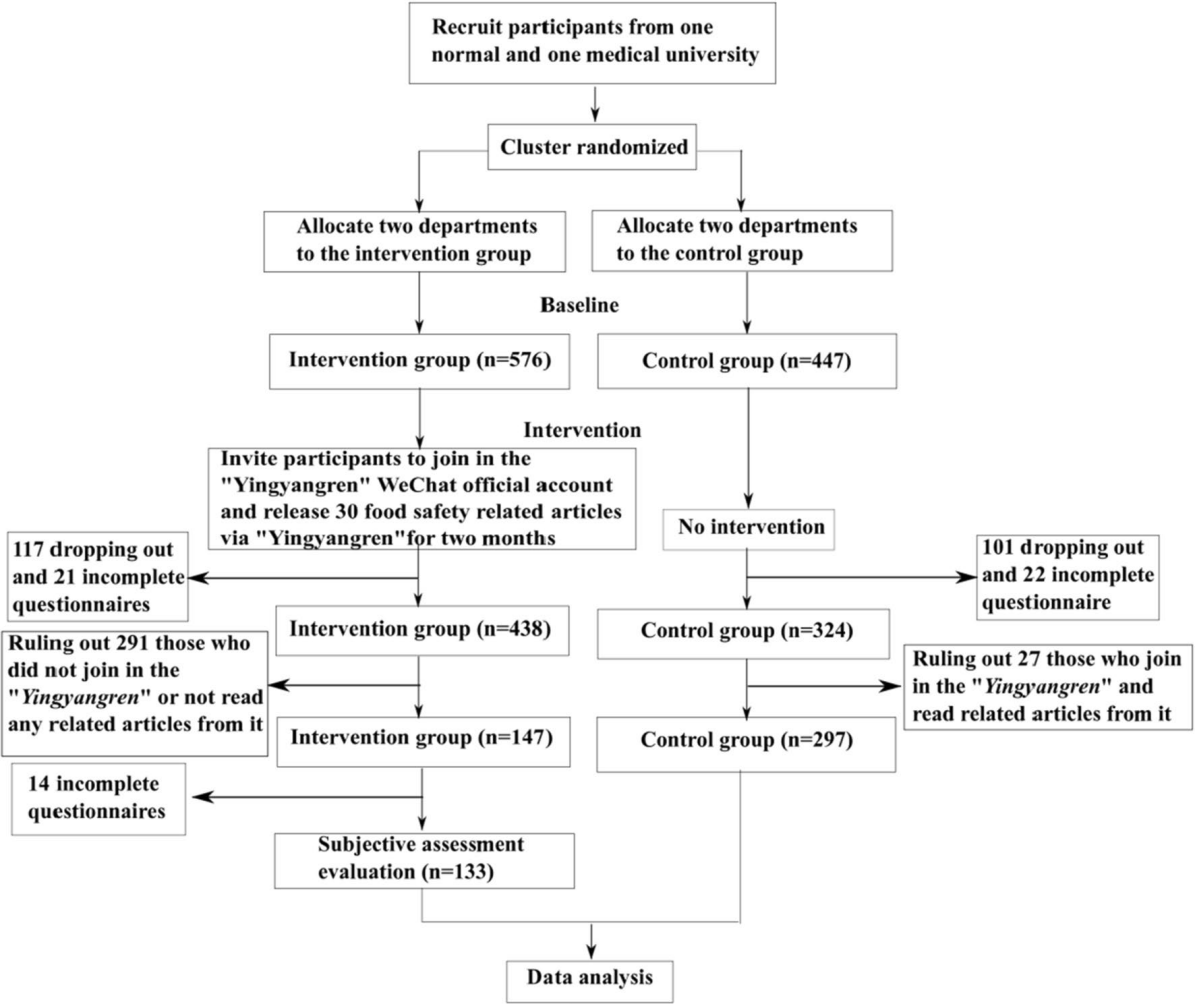
This figure demonstrates the study protocol flowchart of food safety-related health education via WeChat official account

This study was conducted for two months. Educational materials included a total of 30 food safety-related popular science articles on eight themes (three articles per week, on average) to participants in the intervention group. The eight themes were an overview of food safety, foodborne diseases, food labelling, food selection, food preparation, food preservation, food hygiene and others, and each theme included one to four articles written by our research group. Food safety-related popular science articles were released at 21:30 on Monday, Wednesday and Friday by the "Yingyangren" WeChat official account, and re-tweeted via QQ or Micro-blog. At baseline, the intervention group included 576 university students, and the control group included 447 university students. After the intervention, the participants who were lost to follow-up (n = 218) answered the questionnaires incompletely (n = 43), did not join the "Yingyangren" WeChat official account or did not read food safety-related popular science articles in the intervention group (n = 291), and those who joined the "Yingyangren" WeChat official account and read food safety-related popular science articles in the control group (n = 27) were excluded. Thus, in the post-intervention data analysis, the intervention group included 147 university students, and the control group included 297 university students. In addition, owing to the incomplete answers to the questionnaires (n = 14), 133 university students were included in the subjective assessment evaluation analysis after being excluded. The flowchart of this study is shown in Fig. 1.

### Evaluation of intervention effect

The questionnaire on effect evaluation included demographic characteristics, food safety KAP, feedback from reading food safety-related popular science articles, subjective assessment evaluation with the health education programs and the other ways to obtain food safety information in the last two months. The detailed questionnaire is provided in Additional file 1: Table 1.

### Incentive motivation

The previous study demonstrated that incentive motivation increases response rates in health intervention [38]. Therefore, awards were given for answering the related questions correctly to improve the participants’ compliance. A quiz about food safety knowledge related to previously released articles was provided at the end of each article. Students in the intervention group could answer those questions by leaving a message. We would announce the list of participants who responded correctly in the next article, and ‘red envelopes’ (monetary gifts) on WeChat were given to the participants as rewards.

### Measurements and outcomes

Food safety KAP was measured using a self-administrated questionnaire. This questionnaire consisted of 33 questions. A total of 16 single-choice questions were used to measure knowledge (0 = not true or do not know, 1 = true). A total of 10 five-point Likert style (strongly disagree, disagree, neutral, agree and strongly agree) questions were used to measure attitudes (scored 1 to 5). Seven five-point Likert style (never, occasionally, sometimes, often, always) questions were used to measure practices (scored 1 to 5). A low score is regarded as having poor food safety KAP. The internal consistency of the KAP questionnaire was acceptable (Cronbach’s alpha = 0.86). A detailed description of the assessment of food safety KAP has been mentioned [6]. A number of potential confounding factors were included as covariates in the analyses, including age, body mass index, gender (male/female), ethnicity (Han ethnic/Minority), residence (urban/rural), monthly living expenses (< 800RMB/800–1200RMB/ > 1200RMB) and parents’ educational level (low: primary school or below, medium: secondary school, high: high school or secondary vocational school or college or above). These factors were chosen because they were either known or plausible mediating/confounding factors for food safety KAP [6, 7].

### Quality control

All the investigators were recruited via interview to join the investigation team. They were trained uniformly and required to understand the approach, objectives and methodology of this study, as well as being full of experience in handling potentially sensitive issues. The questionnaire was adapted from the previous literature and repeatedly revised through expert interviews. Moreover, team members would communicate with teachers and class leaders in advance to obtain their support and understanding, and they could help us increase students’ active participation. Lastly, the collected questionnaires would be reviewed by investigators to ensure the efficiency of the questionnaire. The data were double-entered in EpiData 3.1 software.

### Statistical analyses

According to the sample size calculation formula $n={({Z}_{1-\alpha /2}+{Z}_{1-\beta})}^{2}\times {2\delta }^{2}/{d}^{2}$, Zx is the x’th percentage point of the standard normal distribution, $d$ represents the difference in treatment means, δ^2^ the total variance in the outcome, n the sample size of each group [39], According to the previous study [40] and assuming a power of 0.80, alpha value of 0.05, it was estimated that 45 participants were needed for each groups. All statistical analyses were performed with STATA (version 12, StataCorp, College Station, Texas, USA). All data were double-checked. Descriptive statistics (frequency or percentages) were used for all variables. χ^2^ tests were used to test statistical differences in the demographic characteristics between the intervention and control groups. An independent t-test was used to test statistical differences in the food safety KAP scores between the two groups. A paired t-test was used to test statistical differences in the food safety KAP scores between before and after the intervention of the two groups. Additionally, quantile regression analysis was conducted to compare the specific quantile of the food safety KAP scores change between the intervention group and the control group after adjusting for sex, ethnicity, residence, expense, education of father and education of mother.

## Results

### Demographic characteristics of participants

Table 1 shows the demographic characteristics of participants and the comparison of the demographic characteristics between the intervention group and the control group. A total of 444 students were included in the final analysis (147 in the intervention group and 297 in the control group). The mean (SD) age of all students was 18.4 (0.76) years, with 32.0% boys and 48.4% living in urban areas. The mean (SD) BMI was 20.4 (2.6). The majority (88.7%) of students were Han ethnic. About half (50.7%) of students’ monthly living expenses was 800–1200 RMB. Education level of students’ fathers accounting for the largest proportion (46.4%) was high school or secondary vocational school or college or above, and secondary school was the largest proportion (36.5%) for their mothers.

**Table 1:** Comparison of the demographic characteristics between the intervention group and the control group

Variables		Total (n = 444)	Intervention group (n = 147)	Control group (n = 297)	*p*
Gender (n, %)	Male	142 (32.0)	49 (33.3)	93 (31.3)	0.67
	Female	302 (68.0)	98 (66.7)	204 (68.7)	
Age (Mean ± SD)		18.4 (0.76)	18.4 (0.8)	18.4 (0.7)	0.89
BMI (Mean ± SD)		20.4 (2.6)	20.4 (2.6)	20.4 (2.6)	0.99
Ethnic category (n, %)	Han ethnic	394 (88.7)	133 (90.5)	261 (87.9)	0.42
	Minority	50 (11.3)	14 (9.5)	36 (12.1)	
Residence (n, %)	Urban	215 (48.4)	74 (50.3)	141 (47.5)	0.57
	Rural	229 (51.6)	73 (49.7)	156 (52.5)	
Monthly living expenses	< 800	76 (17.1)	32 (21.8)	44 (14.8)	0.081
(RMB)	800–1200	225 (50.7)	76 (51.7)	149 (50.2)	
	> 1200	143 (32.2)	39 (26.5)	104 (35.0)	
Father’s educational level	Low	78 (17.6)	28 (19.0)	50 (16.8)	0.85
(n, %)	Medium	160 (36.0)	52 (35.4)	108 (36.4)	
	High	206 (46.4)	67 (45.6)	139 (46.8)	
Mother’s educational level	Low	128 (28.8)	44 (29.9)	84 (28.3)	0.90
(n, %)	Medium	162 (36.5)	54 (36.7)	108 (36.4)	
	High	154 (34.7)	49 (33.3)	105 (35.4)	

### Comparison of food safety KAP score between the two groups before and after intervention

Table 2 shows the comparison of the mean (SD) food safety KAP score between the two groups before and after intervention (before intervention: 10.7 ± 2.1 vs 10.4 ± 2.3 for knowledge (*p* = 0.22), 40.0 ± 4.7 vs 38.9 ± 5.1 for attitude (*p* = 0.03), and 27.2 ± 3.9 vs 26.6 ± 4.2 for practice (*p* = 0.17); after intervention: 11.2 ± 2.1 vs 11.2 ± 2.4 for knowledge (*p* = 0.98), 39.3 ± 5.3 vs 38.4 ± 5.4 for attitude (*p* = 0.13), and 28.2 ± 4.2 vs 27.7 ± 4.4 for practice (*p* = 0.21)).

**Table 2:** Comparison of students’ food safety KAP scores between the two groups before and after the intervention

Variables	Before intervention (mean ± SD)			After intervention (mean ± SD)		
	Intervention group (n = 147)	Control group (n = 297)	*p*	Intervention group (n = 147)	Control group (n = 297)	*p*
Knowledge	10.7 ± 2.1	10.4 ± 2.3	0.22	11.2 ± 2.1	11.2 ± 2.4	0.98
Attitudes	40.0 ± 4.7	38.9 ± 5.1	0.03	39.3 ± 5.3	38.4 ± 5.4	0.13
Practices	27.2 ± 3.9	26.6 ± 4.2	0.17	28.2 ± 4.2	27.7 ± 4.4	0.21

### Comparison of food safety KAP score of the two groups between before and after intervention

Table 3 shows the comparison of the mean (SD) food safety KAP score of the two groups between before and after intervention (the intervention group: 10.7 ± 2.1 vs 11.2 ± 2.1 for knowledge (*p* = 0.01), 40.0 ± 4.7 vs 39.3 ± 5.3 for attitude (*p* = 0.07), and 27.2 ± 3.9 vs 28.2 ± 4.2 for practice (*p* = 0.01); the control group: 10.4 ± 2.3 vs 11.2 ± 2.4 for knowledge (*p* = 0.0003), 38.9 ± 5.1 vs 38.4 ± 5.4 for attitude (*p* = 0.11), and 26.6 ± 4.2 vs 27.7 ± 4.4 for practice (*p* = 0.0001)).

**Table 3:** Comparison of students’ food safety KAP scores between before and after the intervention among the two groups

Variables	Intervention group (n = 147) (mean ± SD)			Control group (n = 297) (mean ± SD)		
	Before intervention	After intervention	*p*	Before intervention	After intervention	*p*
Knowledge	10.7 ± 2.1	11.2 ± 2.1	0.01	10.4 ± 2.3	11.2 ± 2.4	0.0003
Attitudes	40.0 ± 4.7	39.3 ± 5.3	0.07	38.9 ± 5.1	38.4 ± 5.4	0.11
Practices	27.2 ± 3.9	28.2 ± 4.2	0.01	26.6 ± 4.2	27.7 ± 4.4	0.0001

### Feedback from reading food safety-related popular science articles

The reading rate of each popular science article of is shown in Additional file 1: Table 2. Additional file 1: Table 3 shows that 66.9% of the students obtained these popular science articles from the "Yingyangren" WeChat official account. 74.4% of the students read the full text roughly, only 11.3% read the full text carefully when browsing the popular science articles. Approximately half of the participants showed appreciation or used bookmarks while reading the popular science articles of interest. However, 18.8% of the students would do nothing. Text combined with images was the favourite type of popular science articles amongst the participants.

### Subjective assessment evaluation of food safety education intervention program

Additional file 1: Table 4 shows that 63.2% of the students liked these popular science articles with a storyline. 73.7% of the students thought these popular science articles were partially understandable, and 91.7% and 94.7% of the students thought the articles provided new information on food safety and were directly related to their daily life. 72.9%, 69.2% and 67.7% of the students thought that these released food safety-related popular science articles were trustworthy, can improve their food safety knowledge and correct their inappropriate behaviours, respectively.

### Other ways to obtain food safety-related information

Regarding the other ways to obtain food safety information amongst the participants in the last two months, Additional file 1: Table 5 shows that other social media or network platforms, classmates or friends, television or newspaper accounted for 56.8%, 36.5%, and 34.0%, respectively.

### Quantile regression analysis

**Fig. 2:**
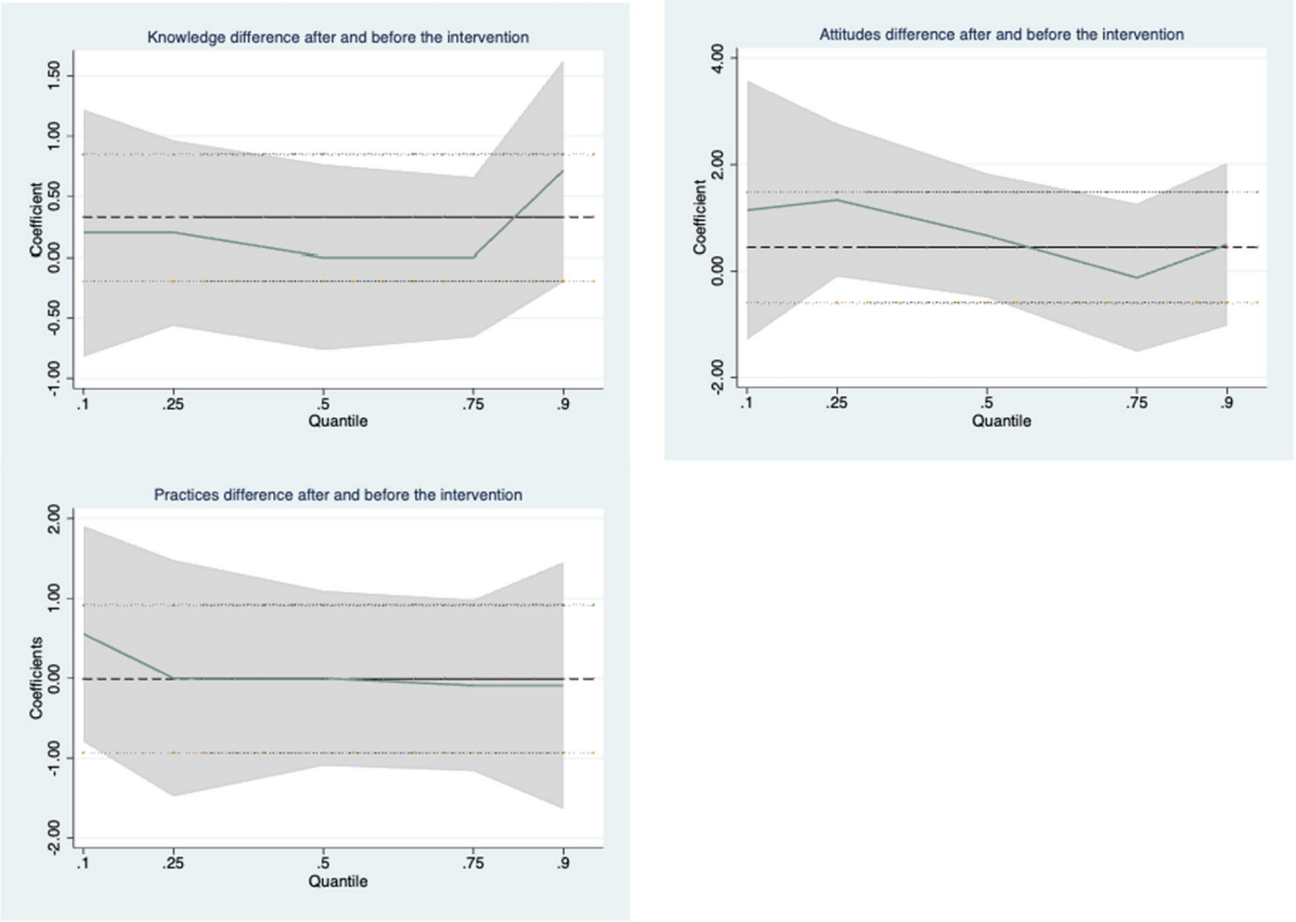
Coefficients (β) for the associations of food safety KAP change associated with intervention across the quantile levels of KAP change

Quantile regression analysis shows that the intervention had no effects on food safety KAP improvement amongst the participants after adjusting sex, ethnicity, residence, expenses and education of father and mother. The β-coefficients [95% confidence intervals (CI)] for the 10th, 50th and 90th percentiles were 0.2 [− 0.86, 1.26], 0.00 [− 0.54, 0.54], 0.71 [− 0.14, 1.57] for knowledge, 1.14 [− 0.25, 2.53], 0.67 [− 1.02, 2.35] and 0.50 [− 1.27, 2.27] for attitudes, 0.56 [− 0.80, 1.91], 0.00 [− 1.02, 1.02] and − 0.09 [− 2.07, 1.89] for practices. The results are shown in Fig. 2.

## Discussion

Results of this study showed that no improvement was found in food safety KAP scores of university students between the intervention and control groups after the intervention. These results are similar to the results of a systematic review and meta-analysis of randomized controlled trials for diet and exercise behaviour interventions through social media [32], which concluded no significant differences between groups in key outcomes. The following possible factors for limited intervention effects in this study should be considered. Firstly, university students had a relatively low level of personal involvement in food safety [41, 42]. Though they are considered as one of the high-risk population groups for food poisoning, food safety is not a priority subject when they think about food [42]. Another reason for the low level of involvement may be the acceptance of types of online information; university students were active recipients for social networking sites, particularly for communication purposes; however, they appeared to accept knowledge or health information passively via the Internet [43–45]. Taking the initiative to read the food safety-related popular science articles released through the WeChat official account amongst the intervention group might be a great challenge. Although we offered financial incentives and various types of intervention materials (e.g. text, images, audio and video) to attract additional interest and willingness to engage in this study, and we strive to obtain teachers’ and class leaders’ support so that they can encourage students to participate, the low levels of participation were still observed. In the intervention group, approximately two-thirds of the participants did not join the "Yingyangren" WeChat official account or read any articles from it. Moreover, this study found a low rate of reading food safety-related popular science articles. Medical university school is a stressful environment due to the extensive curricula, numerous academic requirements and frequent, difficult and various types of examinations [46, 47], and medical students may have no time and energy to participate in this intervention. Thus, new methods to mobilize the enthusiasm and increase the participation of university students should be explored and attempted in a future intervention study, such as sending links or emails to invite participants to view relevant content [48]. In addition, the duration of social media intervention should avoid the period when university students would be busy with many examinations.

Secondly, part of intervention information through articles published in "Yingyangren" WeChat official account may not be appropriate for university students. "Yingyangren" WeChat official account is a relatively experienced platform for delivering health knowledge, university students in this intervention are a part of this official account followers. Some contents of the articles provided are related to popular issues to attract interest of official account followers, but the relevance to university students is under considered, such as the veterinary drug residue of meat and pesticide residues in vegetables; these topic can be hardly put into practice by the Chinese university students who mostly live a campus life. Information intervention may be invalid. WeChat official account should be set up specifically in future intervention for target population audience. In addition, KAP model was used to evaluate the intervention effects in this study. In order to ensure the reliability and validity of the KAP questionnaire, the items of questionnaire were selected. Hence, the final questions used to measure the intervention effects were not exactly kept in line with the intervention materials/science articles; this could be the one of the reasons for the limited intervention effects.

Thirdly, diversified health information acquisition amongst university students [49] may be another reason for the limited effects. In this study, popular science articles released by the "Yingyangren" WeChat official account would be re-tweeted via WeChat moment, QQ or Micro-blog to increase accessibility amongst the intervention group. However, during the process, the participants in the control group may also obtain the intervention information indirectly. Moreover, results of this study showed that more than half of the participants in the control group obtained food safety-related knowledge via other social media or network platforms. In addition to the provided intervention platform and information, the participants can also obtain food safety-related information through other channels, which may cause a considerable improvement in food safety knowledge and practices in the intervention and control groups after the intervention and the no difference between-group findings in this study.

Intervention strategies of social media could enhance the success rate, such as the integrated use of discussion boards, learning modules, tailored feedback and interactivity [50, 51]. However, in this study, the WeChat official account was used to release food safety-related popular science articles, and learning modules were mainly intervention strategies for participants, whilst other functions of WeChat are not utilized efficiently in this study. This factor may be considered in analysing the limited intervention effects. In future studies, discussion boards, tailored feedback and interactivity of the WeChat official account should be utilized efficiently to enhance the success rate. Moreover, using social media as part of a complex intervention, which can combine the WeChat official account for online food safety education and offline lectures or food safety-related compulsory courses, could be conducted amongst university students.

The results of this study showed that intervention materials had a certain degree of readability and effectiveness. In addition, the participants had a relatively high level of satisfaction with the "Yingyangren" WeChat official account for conducting food safety education intervention. Most participants trusted the food safety-related popular science articles released by "Yingyangren" WeChat official account and agreed that the information could help to improve their food safety knowledge and correct their inappropriate behaviours. However, subjective assessment was not in accordance with the intervention results. The possible explanation for the inconsistent results could be that university students is relatively optimistic and may exaggerate the effects intervention, and the questions used to measure the intervention effects is somewhat difficult for them. Additional studies on how to efficiently use the WeChat official account to improve the food safety knowledge and correct inappropriate food safety behaviours of university students should be conducted.

This study has certain limitations. Firstly, this study did not design a targeted educational program for the student audience, and "Yingyangren" WeChat official account was not specifically established for this intervention group; part of intervention information may not be appropriate for university students; the KAP questionnaire was not exactly kept in line with the intervention materials. Future intervention by social media needs to be strengthened in these three aspects. Secondly, the intervention duration might not be enough. Practice change needs regular long-term education. One systematic review showed that the duration of social media intervention ranged from three months to two years [32]. However, the duration of this study was two months. The study duration could be increased to examine the intervention effects in future studies. Moreover, evaluating the food safety KAP during the two-month intervention process should be considered instead of just evaluating the KAP before and after the intervention, such as conducting an assessment after completing the three or four-time interventions. Thirdly, interaction characteristics in social media are one of the most common features [32, 52, 53], such as message boards and consulting section in this study. However, very few participants expressed their own opinions or raised questions about released food safety-related popular science articles. How to efficiently use the essential interaction characteristics should also be studied. Fourthly, this study relied on self-report, which may introduce bias caused by dishonesty, measurement flaws or social desirability bias. Fifthly, our intervention was implemented in small sample. Generalizability to larger units would be necessary. Finally, usage of Internet interventions was typically low, and high attrition rates are one of the possible reasons [32]. Similarly, high attrition rate in this study could introduce bias into the results, although no difference exists in the socio-demographic characteristics between the intervention and control groups before and after the intervention. Challenges of adherence and keeping the participants engaged, incentive motivation and end-user engagement during the development of the intervention could be attempted in future research to decrease the attrition [54, 55].

## Conclusion

The WeChat official account intervention had a limited effect on improving the food safety KAP amongst university students. This study was an exploration of food safety intervention using the WeChat official account; valuable experience can be provided for social media intervention in future study. Given that university students are the key population for food safety intervention and social media has become the main method for them to obtain information, powerful trials and meta-analyses are required to explore how to efficiently use the WeChat official account intervention on food safety health education and how to improve the intervention effects in future studies.

## Supplementary Information

**Additional file 1: Figure 1.** Introduction of “Yingyangren” WeChat official account. **Table 1.** The questionnaire on effect evaluation of WeChat based intervention on food safety KAP among university students. **Table 2.** The reading rate of each popular science article released by “Yingyangren” WeChat official account in the intervention group. **Table 3.** Feedback from reading food safety-related popular science articles released by “Yingyangren” WeChat official account in the intervention group. **Table 4.** Subjective assessment evaluation with the food safety-related popular science articles released by “Yingyangren” WeChat official account in the intervention group. **Table 5.** Other ways to obtain food safety-related information among all university students.

## Data Availability

Data set underlying the findings are available from the corresponding author on reasonable request.

## Abbreviations

KAP: Knowledge, attitudes and practices
