# Supplementary material for: Effect of WeChat-based intervention on food safety knowledge, attitudes and practices among university students in Chongqing, China: a quasi-experimental study
Source: J Health Popul Nutr. 2023 Apr 5;42:28. doi: 10.1186/s41043-023-00360-y (PMC10074872; doi:10.1186/s41043-023-00360-y)
Supplement: Supplementary file 1 — Additional file 1: Figure 1. Introduction of “Yingyangren” WeChat official account. Table 1. The questionnaire on effect evaluation of WeChat based intervention on food safety KAP among university students. Table 2. The reading rate of each popular science article released by “Yingyangren” WeChat official account in the intervention group. Table 3. Feedback from reading food safety-related popular science articles released by “Yingyangren” WeChat official account in the intervention group. Table 4. Subjective assessment evaluation with the food safety-related popular science articles released by “Yingyangren” WeChat official account in the intervention group. Table 5. Other ways to obtain food safety-related information among all university students. [file 41043_2023_360_MOESM1_ESM.docx]

**Supplementary figure 1.** Introduction of "Yingyangren" WeChat official account


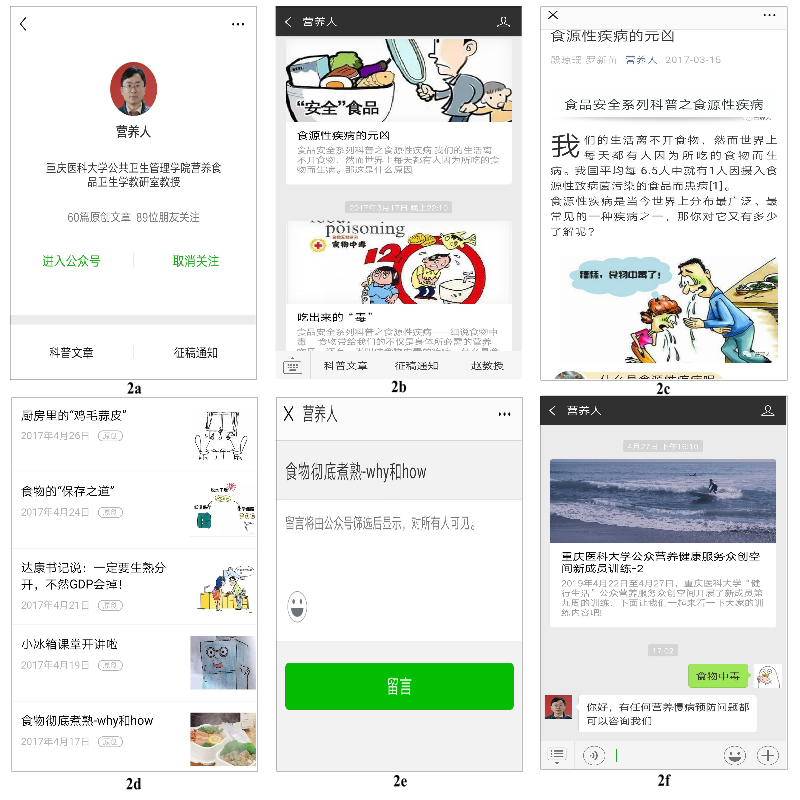


1a Profile of the "Yingyangren" WeChat official account; 1b profile of the message sending page; 1c profile of the message reading page; 1d Profile of the consulting page; 1e Profile of messages leaving rage; 1f Profile of messages leaving rage.

**Supplementary table 1.** The questionnaire on effect evaluation of WeChat based intervention on food safety KAP among university students

| Variables | Questions |
| --- | --- |
| Demographic characteristics | The part of demographic characteristics of participants was the same with the baseline questionnaire. |
| Food safety KAP | The part of food safety KAP questionnaire was the same with the baseline questionnaire. |
| Feedback from reading food safety-related popular science articles | 1. Have you read the following articles (30 articles in total)? (yes / no) |
|  | 1. Which platforms do you get those popular science articles? ("Yingyangren" WeChat official account, WeChat group, WeChat moments, QQ group, QQ moments, Micro-blog, others) |
|  | 1. How do you browse those popular science articles? (only read the title, read selective paragraphs, roughly read the full text, carefully read the full text) |
|  | 1. What would you do while reading popular science articles of interest? (re-tweeted or share, show appreciation, leave messages, bookmark, give reward, discuss with others, do nothing) |
|  | 1. What kind of popular science articles do you like most? (text and images, text, images and audio, text, images and video) |
| Subjective assessment evaluation with the health education program. | 1. Do you like those popular science articles with a storyline released by "Yingyangren" WeChat official account? (a lot, not a lot but like, just like, dislike, hate it) |
|  | 1. Can you understand those popular science articles released by "Yingyangren" WeChat official account? (fully understandable, partially understandable, completely incomprehensible) |
|  | 1. I think that those popular science articles are directly related to my daily life. (yes/no) |
|  | 1. I trust those popular science articles released by "Yingyangren" WeChat official account. (strongly agree, agree, neutral, disagree, strongly disagree) |
|  | 1. The popular science articles help me improve food safety knowledge. (strongly agree, agree, neutral, disagree, strongly disagree) |
|  | 1. The message helps me correct inappropriate behaviors. (strongly agree, agree, neutral, disagree, strongly disagree) |
| Other ways to obtain food safety-related information | 1. What other ways do you gain food safety knowledge in the last two months? (food safety-related curriculum, other social media or network platform, parents telling, classmates or friends telling, TV or newspaper, others) |

**Supplement table 2.** The reading rate of each popular science article released by "Yingyangren" WeChat official account in the intervention group

| Title of popular science articles | Yes (n, %) | No (n, %) | Forgot (n, %) |
| --- | --- | --- | --- |
| Food safety on the tip of the tongue. | 94 (70.7) | 26 (19.5) | 13 (9.8) |
| “Murderer” of foodborne disease. | 71 (53.4) | 44 (33.1) | 18 (13.5) |
| Talking about food poisoning. | 85 (63.9) | 35 (26.3) | 13 (9.7) |
| [The food produced in nuclear radiation areas](http://www.ncbi.nlm.nih.gov/pubmed/20525606). | 60 (45.1) | 56 (42.1) | 17 (12.8) |
| Food allergy. | 54 (40.6) | 60 (45.1) | 19 (14.3) |
| [H7N9 bird flu](http://xueshu.baidu.com/s?wd=paperuri:(6c7774ab37861490f86d4c9666d61ec9)&filter=sc_long_sign&sc_ks_para=q=Structural%20aberration%20in%20R282K%20genetic%20mutation%20and%20antiviral%20drug-resistant%20H7N9%20bird%20flu&sc_us=13636275920069960224&tn=SE_baiduxueshulib_9r82kicg&ie=utf-8&sc_as_para=sc_lib:cqmu&sc_from=cqmu) “bombing”, please check your “equipment”. | 78 (58.6) | 44 (33.1) | 11 (8.3) |
| Environmental pollution, is food safe? | 75 (56.4) | 41 (30.8) | 17 (12.8) |
| Risk of food poisoning. | 70 (52.6) | 49 (36.8) | 14 (10.5) |
| Food label, do you understand it? | 80 (60.2) | 35 (26.3) | 18 (13.5) |
| Nutrition label: “eat clearly”. | 74 (55.6) | 45 (33.8) | 14 (10.5) |
| Strategy for purchasing pre-packaged food. | 69 (51.9) | 47 (35.3) | 17 (12.8) |
| Talking about the seafood we loved. | 66 (49.6) | 57 (42.9) | 10 (7.5) |
| Online shopping, be careful. | 84 (63.2) | 33 (24.8) | 16 (12.0) |
| Taking about [the safety of imported foods](http://xueshu.baidu.com/s?wd=paperuri:(8e584e7dd1340332700e75cb5b913af1)&filter=sc_long_sign&sc_ks_para=q=Improving%20the%20Safety%20of%20Imported%20Foods%20With%20Intelligent%20Systems&sc_us=8841875622419074660&tn=SE_baiduxueshu_c1gjeupa&ie=utf-8). | 62 (46.6) | 47 (35.3) | 24 (18.0) |
| How to choose eggs? | 72 (54.1) | 47 (35.3) | 14 (10.5) |
| Do you purchase the qualified food? | 70 (52.6) | 52 (39.1) | 11 (8.3) |
| Cooking food thoroughly, why and how? | 72 (54.1) | 51 (38.3) | 10 (7.5) |
| Store food correctly within your refrigerators. | 69 (51.9) | 48 (36.1) | 16 (12.0) |
| Raw foods and cooked foods should separate. | 70 (52.6) | 40 (30.1) | 23 (17.3) |
| [Food storing.](http://xueshu.baidu.com/s?wd=paperuri:(ca44eed0e1de964539895fcd127d1f1f)&filter=sc_long_sign&sc_ks_para=q=Food%20storing%20by%20marsh%20tits&sc_us=14705148734920325847&tn=SE_baiduxueshu_c1gjeupa&ie=utf-8) | 81 (60.9) | 40 (30.1) | 12 (9.0) |
| [The importance of hygiene in the kitchen.](http://www.cabdirect.org/abstracts/20093105226.html) | 74 (55.6) | 47(35.3) | 12(9.0) |
| Illness food its way in by the mouth, how to wash your hands correctly. | 82 (61.7) | 36 (27.1) | 15 (11.3) |
| Talking about eating out. | 77 (57.9) | 45 (33.8) | 11 (8.3) |
| [New Food Safety Law of China and consumers' right.](http://xueshu.baidu.com/s?wd=paperuri:(b459a9fb07d3b4d55747e66b1e526871)&filter=sc_long_sign&sc_ks_para=q=New%20Food%20Safety%20Law%20of%20China%20and%20the%20special%20issue%20on%20food%20safety%20in%20China&sc_us=8048868209727176682&tn=SE_baiduxueshu_c1gjeupa&ie=utf-8) | 60 (45.1) | 58 (43.6) | 15 (11.3) |
| The latest domestic food safety news | 64 (48.1) | 47(35.3) | 22 (16.5) |
| Talking about “junk food” | 79 (59.4) | 43 (32.2) | 11 (8.3) |
| Self-report of “food additives”. | 68 (51.1) | 46 (34.6) | 19 (14.3) |
| Microbial contamination. | 66 (49.6) | 49 (36.8) | 18 (13.5) |
| Say no to [chemical contaminants in food](http://xueshu.baidu.com/s?wd=paperuri:(7ffc951edb99415fe87481f4216e4008)&filter=sc_long_sign&sc_ks_para=q=Liquid%20chromatography-mass%20spectrometry%20for%20the%20determination%20of%20chemical%20contaminants%20in%20food&sc_us=3170792863519293924&tn=SE_baiduxueshu_c1gjeupa&ie=utf-8). | 56 (42.1) | 60 (45.1) | 17 (12.8) |
| Talking about physical contamination in food. | 67 (50.4) | 53 (39.8) | 13 (9.8) |

**Supplement table 3.** Feedback from reading food safety-related popular science articles released by "Yingyangren" WeChat official account in the intervention group

| Variables | n (%) |
| --- | --- |
| Which platforms do you get these popular science articles? |  |
| "Yingyangren" WeChat official account | 89 (66.9) |
| WeChat group | 51 (38.3) |
| WeChat Moment | 36 (27.1) |
| QQ group | 28 (21.1) |
| QQ Moment | 12 (9.0) |
| Micro-blog | 15 (11.3) |
| Others | 6 (4.5) |
| How do you browse these popular science articles? |  |
| Only read title | 23 (17.3) |
| Read selective paragraph | 45 (33.8) |
| Roughly read the full text | 103 (74.4) |
| Carefully read the full text | 15 (11.3) |
| What would you do while reading popular science articles of interest? |  |
| Re-tweeted or share | 41 (30.8) |
| Show appreciation | 71 (53.4) |
| Leave messages | 11 (8.3) |
| Bookmark | 67 (50.4) |
| Give reward | 11 (8.3) |
| Discuss with others | 13 (9.8) |
| Do nothing | 25 (18.8) |
| What kind of type of popular science articles do you like most? |  |
| Text combined with images | 61(45.9) |
| [Text combined with images](http://xueshu.baidu.com/s?wd=paperuri:(11f3021401c202f32895c84dc3da6e97)&filter=sc_long_sign&sc_ks_para=q=Selling%20Digital%20Rights%20as%20a%20General%20Publisher:%20Text%20and%20Images&sc_us=15762374374335401291&tn=SE_baiduxueshu_c1gjeupa&ie=utf-8) and audio | 48(36.1) |
| [Text combined with images](http://xueshu.baidu.com/s?wd=paperuri:(11f3021401c202f32895c84dc3da6e97)&filter=sc_long_sign&sc_ks_para=q=Selling%20Digital%20Rights%20as%20a%20General%20Publisher:%20Text%20and%20Images&sc_us=15762374374335401291&tn=SE_baiduxueshu_c1gjeupa&ie=utf-8) and video | 56 (42.1) |

**Supplement table 4.** Subjective assessment evaluation with the food safety-related popular science articles released by "Yingyangren" WeChat official account in the intervention group

| Subjective assessment evaluation | n (%) |
| --- | --- |
| Do you like these popular science articles with a storyline released by "Yingyangren" WeChat official account? |  |
| A lot | 9 (6.8) |
| Not a lot but like | 75 (56.4) |
| Just like | 46 (34.6) |
| Dislike | 3 (2.2) |
| Hate it | 0 (0.0) |
| Can you understand these popular science articles released by "Yingyangren" WeChat official account? |  |
| Fully understandable | 31 (23.3) |
| Partially understandable | 98 (73.7) |
| Completely incomprehensible | 4 (3.0) |
| I think that these popular science articles provided new information about food safety. |  |
| Yes | 122 (91.7) |
| No | 11 (8.3) |
| I think that these popular science articles are directly related to my daily life. |  |
| Yes | 126 (94.7) |
| No | 7 (5.3) |
| I trust these popular science articles released by "Yingyangren" WeChat official account. |  |
| Strongly agree | 25 (18.8) |
| Agree | 72 (54.1) |
| Neutral | 34 (25.6) |
| Disagree | 2 (1.5) |
| Strongly disagree | 0 (0) |
| The popular science articles help me improve food safety knowledge. |  |
| Strongly agree | 12 (9.0) |
| Agree | 80 (60.2) |
| Neutral | 35 (26.3) |
| Disagree | 6 (4.5) |
| Strongly disagree | 0 (0) |
| The popular science articles help me correct inappropriate behaviors. |  |
| Strongly agree | 17 (12.8) |
| Agree | 73 (54.9) |
| Neutral | 41 (30.8) |
| Disagree | 2 (1.5) |
| Strongly disagree | 0 (0) |

**Supplement table 5.** Other ways to obtain food safety-related information among all university students

| What other ways do you gain food safety knowledge in the last two months? | | Total (n = 444) | Intervention group (n = 147) | Control group (n = 297) |
| --- | --- | --- | --- | --- |
| Food safety-related curriculum | 58 (13.1) | 35 (23.8) | 23 (7.7) |  |
| Food safety-related lecture | 55 (12.4) | 41 (27.9) | 14 (4.7) |  |
| Other social media or network platform | 252 (56.8) | 92 (62.6) | 160 (53.9) |  |
| Parents | 123 (27.7) | 36 (24.5) | 87 (29.3) |  |
| Classmates or friends | 162 (36.5) | 55 (37.4) | 107 (36.0) |  |
| TV or newspaper | 151 (34.0) | 51 (34.7) | 100 (33.7) |  |
| Other ways | 13 (2.9) | 13 (8.8) | 36 (12.1) |  |
